# Effects of High-Intensity Resistance Training on Physical Fitness, Hormonal and Antioxidant Factors: A Randomized Controlled Study Conducted on Young Adult Male Soccer Players

**DOI:** 10.3390/biology11060909

**Published:** 2022-06-13

**Authors:** Ana Filipa Silva, Mohammad Hosein Aghidemand, Masoud Kharatzadeh, Vahab Khan Ahmadi, Rafael Oliveira, Filipe Manuel Clemente, Georgian Badicu, Eugenia Murawska-Ciałowicz

**Affiliations:** 1 Escola Superior Desporto e Lazer, Instituto Politécnico de Viana do Castelo, Rua Escola Industrial e Comercial de Nun’Álvares, 4900-347 Viana do Castelo, Portugal; anafilsilva@gmail.com; 2 Research Center in Sports Performance, Recreation, Innovation and Technology (SPRINT), 4960-320 Melgaço, Portugal; 3 The Research Centre in Sports Sciences, Health Sciences and Human Development (CIDESD), 5001-801 Vila Real, Portugal; rafaeloliveira@esdrm.ipsantarem.pt; 4 Department of Exercise Physiology, Faculty of Sport Sciences, Allameh Tabatabai University, Q756+R4F Dehkadeh-ye-Olympic, Tehran 14896-84511, Iran; mohammadhoseinaghidemand@gmail.com (M.H.A.); vahab.khanahmadi@gmail.com (V.K.A.); 5 Sports Scientist, Sepahan Football Club, Isfahan 81887-78473, Iran; masoud.kharatzadeh@gmail.com; 6 Department of Exercise Physiology, Faculty of Sport Sciences, University of Isfahan, Isfahan 81746-7344, Iran; 7 Sports Science School of Rio Maior, Polytechnic Institute of Santarém, 2040-413 Rio Maior, Portugal; 8 Life Quality Research Centre, 2040-413 Rio Maior, Portugal; 9 Instituto de Telecomunicações, Delegação da Covilhã, 1049-001 Lisboa, Portugal; 10 Department of Physical Education and Special Motricity, University Transilvania of Brasov, 500068 Brasov, Romania; georgian.badicu@unitbv.ro; 11 Department of Physiology and Biochemistry, University School of Physical Education, 51-612 Wrocław, Poland; eugenia.murawska-cialowicz@awf.wroc.pl

**Keywords:** football, aerobic fitness, hormones, physical fitness, strength, exercise

## Abstract

**Simple Summary:**

We aimed to analyze the effects of high-intensity resistance training (HIRT) on physical fitness and hormonal and antioxidant factors of adult young male soccer players. We have compared an 8-week intervention using HIRT period with a control group that only performed regular technical/tactical sessions. The soccer players were assessed before and after the intervention for their physical fitness, and hormonal and antioxidant factors. We have found that using HIRT for 8 weeks, with three sessions a week, results in significant improvements in aerobic capacity, sprint at short (10 m) and medium (20 and 30 m) distances, repeated sprint ability, and change-of-direction. This suggests that HIRT combined with regular field-based practice can provide an additional benefit to physical fitness development. Additionally, the group enrolled on HIRT also exhibited significantly decreased levels of cortisol and malondialdehyde, whereas it exhibited significantly increased growth hormone, testosterone, dis-mutase, and glutathione. The results suggest that HIRT can be an interesting approach for improving physical fitness and adjusting the hormonal and antioxidant levels of adult male soccer players.

**Abstract:**

Purpose: The aim of this study was to test the effects of high-intensity resistance training (HIRT) intervention on the physical fitness, hormonal and antioxidant factors of adult male soccer players. Methods: A randomized controlled study design was implemented. Eighteen soccer players (age: 20.3 ± 0.66 years; stature: 174.0 ± 6.01 cm; body mass: 69.1 ± 6.4 kg; body mass index: 22.8 ± 1.6 kg/m^2^) voluntarily participated in this study. Players were assessed before and after an intervention lasting 8 weeks, with three training sessions a week. Assessments of physical fitness included the Yo-Yo intermittent recovery test level 1 (YYIRT1), 10-, 20-, and 30 m sprint time (ST), running-based anaerobic sprint test (RAST) and change-of-direction time (COD). Hormonal tests included cortisol, testosterone and growth hormone (GH), whereas the antioxidant assessment included superoxide dismutase (SOD), malondialdehyde (MDA) and glutathione (GSH). Results: Between-group analysis revealed no significant differences at baseline, whereas it revealed that HIRT presented significant better results than the control group on YYIRT (*p* = 0.032), 10 m ST (*p* = 0.041), 20 m ST (*p* = 0.040), 30 m ST (*p* = 0.044), RAST (*p* = 0.013), and COD (*p* = 0.031) after the intervention period. The within group analysis revealed that the HIRT group significantly improved the YYIRT1 (*p* < 0.001), VO2max (*p* < 0.001), 10 m ST (*p* < 0.001), 20 m ST (*p* = 0.006), 30 m ST (*p* < 0.001), RAST (*p* < 0.001) and COD (*p* < 0.001). Moreover, HIRT group significantly reduced the cortisol (*p* < 0.001) and MDA (*p* = 0.021), whereas it significantly increased the GH (*p* < 0.001), testosterone (*p* < 0.001), SOD (*p* = 0.009) and GSH (*p* = 0.005). Conclusions: The HIRT is effective for improving physical fitness, revealing significant better adaptations than controls. Moreover, hormonal and antioxidant adaptations are also confirmed after HIRT intervention.

## 1. Introduction

The game soccer is an intermittent exercise combining the integration of mixed energetic systems in which periods of low-to-moderate effort are interspaced by short periods of near to maximum efforts [1]. Usually, players are required to cover between 9 and 14 km in a match [2,3], from those 700 to 1200 m are covered in high-intensity running (>19.8 km/h) [4] and 200 to 450 m in sprinting (>25 km/h) [5]. Besides that, they often perform accelerations and decelerations (~600 of each) in different intensities [2]. These locomotor demands require from the players a well-developed physiological and energetic system as well as physical fitness [6].

Athletic performance in soccer players is multilateral and especially challenging for strength and conditioning coaches [7,8]. As metabolic and energetic support for performance, players are required to hold a good aerobic fitness which is frequently a discriminant between elite and non-elite players [9,10]. Moreover, better aerobic fitness is largely correlated with longer distances covered at different speed thresholds [11,12]. Besides the aerobic fitness, is also important to hold well-developed levels of strength and power [13]. As an example, higher levels of maximal strength are largely correlated with better results in linear sprinting performance and jumping [14], whereas jumping in countermovement is largely correlated with linear and curvilinear sprinting [15,16]. Considering that sprinting is the most prevalent action preceding open-play goals [17], supporting such action is key to soccer performance. In fact, better jumping and sprint performance is a discriminant for those achieving higher levels of competition in soccer [18].

Soccer training is especially challenging since coaches should enhance the multitude of physical qualities as above considered [6]. As an example, great attention to high-intensity interval training has been provided. This type of training provides a metabolic stimulus while producing a neuromuscular impact on the players [19]. Although the well-recognized benefits of different high-intensity interval training types [20] on the conditioning of soccer players [21], the muscular strength and power seems not be the main target of such a training method. For such targets, commonly strength and power training methods are often prescribed in weight room [7]. However, the need to use different methods in different moments may increase the time spent in training, and in specific contexts may not be easy-to-apply.

Aiming to combine benefits of high-intensity interval training and regular resistance training, new training modalities have been proposed as the high-intensity resistance training (HIRT) [22], also known as high-intensity functional training [23]. However, since the term functional is complex, discussible, and eventually does not discriminate against the functionality itself, we will keep the term HIRT since resistance training represents a type of strength-building exercise that requires body muscle to exert force against some form of resistance as defined in National Library of Medicine. HIRT emphasizes multi-join movements recruiting a metabolic stimulus while using muscle strengthening exercises, thus employing compound exercises at different intensities [23,24]. Since the different range of movements, intensities, and locomotor/mechanical stimulus, it is supposed to assist to a variety of physiological and physical adaptations [25,26] after training intervention based on the such training method.

The acute responses to HIRT are typically characterized by heart rate responses above 90% maximum heart rate [27] which means that the training modality is particularly stressful in a metabolic point of view [27]. In fact, a study conducted in amateur soccer players comparing HIRT with traditional strength training showed that both cardiorespiratory and metabolic responses were significantly higher in HIRT [22]. Such a physiological stress, and despite interactions with training regimen variables (e.g., intensity, duration, work-to-rest ratio), seems to elicit positive adaptation on aerobic power [28] independently of regimen used. Moreover, the training characteristics of HIRT seems also to improve anaerobic performance pending adjustments in time of bouts and recovery in between [29]. 

Besides metabolic adaptations, endocrine changes can be observed after HIRT. As acute responses, the testosterone and cortisol will suffer variations based on the type of training regimen [30]. However, as long-term adaptations, testosterone is increased after exposure to HIRT as well as enhancing cortisol and growth hormone (GH) hormones [31]. Moreover, it is also expectable to assist changes in oxidative stress level and antioxidant factors, although few research has been conducted in HIRT. Since one of the elements of metabolic stress is oxidative stress, it seems important to analyze the anabolic (testosterone and GH) and catabolic hormone (cortisol) levels in relation to the oxidative stress level and cellular damage provoked, as well as cellular antioxidant response. As example, GH is highly correlated with lower limb power and aerobic capacity which are key physical fitness determinants for soccer performance [32]. Moreover, greater testosterone levels are also associated with better muscle power and sprint supporting player’s performance [33]. Thus, hormonal changes produced by HIRT can expectably impact players’ performance.

The HIRT can also play a role in muscular strength, power, and endurance. Those capacities can be improved, even considering the possible concurrent effects between endurance and strength [34]. In the specific case of a team sport (handball) a form of HIRT based on the circuit was effective for improving vertical jump performance and maximal strength in movements such as the back squat, while increasing the upper-body, leg, and thigh-muscle volumes [25]. Although the HIRT is characterized for a more endurance-based training, it seems to also enhance muscular strength and power which is important in sports as soccer that requires a multilateral physical fitness. This was indirectly suggested by an experimental study conducted in soccer male players which revealed that an approach of HIRT resulted in positive adaptations in speed outcomes [26].

Although the above-mentioned effects, the research about the physical fitness, hormonal, and antioxidant adaptations to HIRT in team sports, and in particular in soccer, is scarce. Thus, research should be conducted to identify variations occurring after exposure to this type of training. This will help to characterize the benefits or risks of using such a training method in soccer and allow coaches to identify if the method can be used in practical scenarios. Based on those reasons, the aim of this study was to test the effects of high-intensity resistance training (HIRT) intervention on the physical fitness, hormonal, and antioxidant stress of adult male soccer players.

## 2. Materials and Methods

### 2.1. Study Design

**Figure 1:**
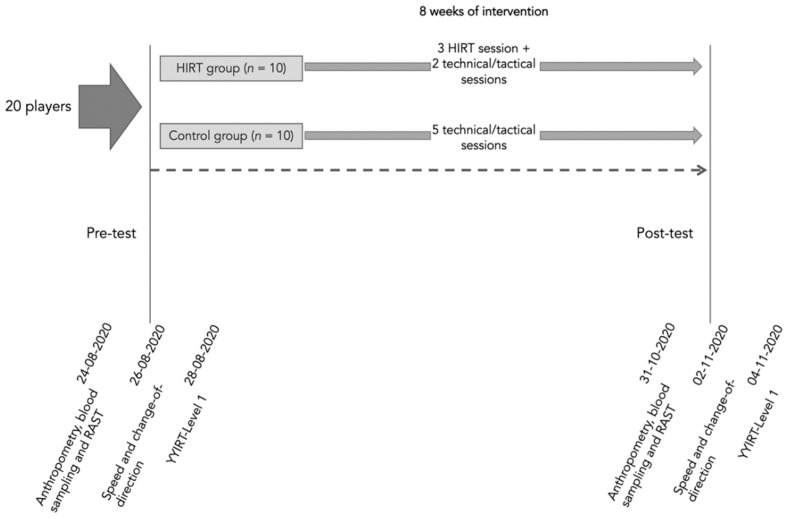
Study overview.

This study employed a randomized controlled design. Convenience sampling was used to selected twenty players from a semi-professional male Iran team (competing in the second league). Simple randomization was implemented to equally distribute players by two groups: HIRT (*n* = 10) and control group (*n* = 10). The study overview can be found in Figure 1.

### 2.2. Setting

The present study was conducted in the early season from 24-08-2020 to 4-11-2020 in the team that were in the second league of Iranian men’s soccer. The training intervention lasted 8 weeks. The weeks before and after the training intervention period were dedicated to physical assessments. The study timeline can be observed in Table 1. 

### 2.3. Participants

**Figure 2:**
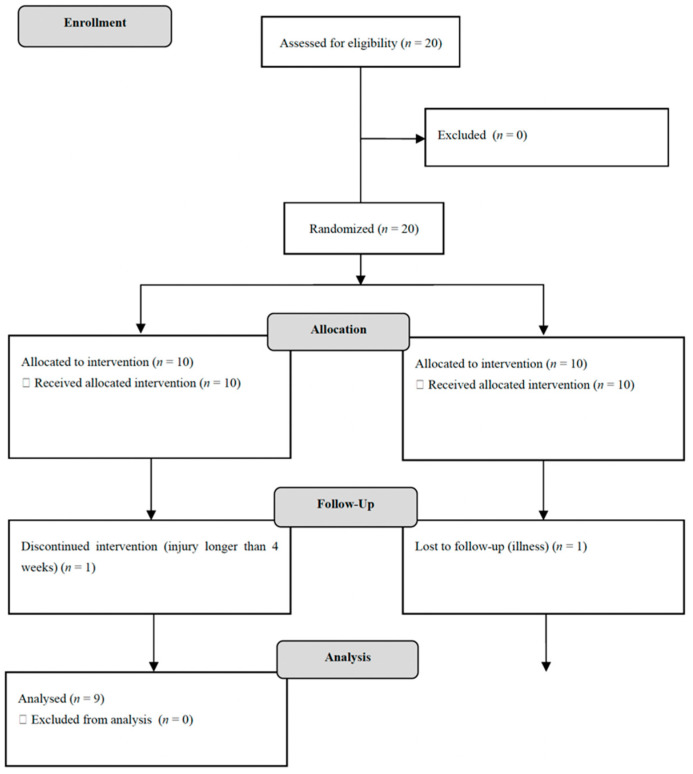
Flow diagram of participants allocation.

An a priori sample size was calculated on G*Power software for a partial eta squared of 0.14 (large effect size, direct option), power of 0.80, correlation among measures of 0.5 and no sphericity correction of 1. There is an 85% chance of correctly rejecting the null hypothesis of no significant effect of the interaction with 8 players HIRT and 8 players control for a total of 16 participants. Although twenty adult male soccer players were enrolled in this study, only eighteen soccer players (age: 20.3 ± 0.66 years; stature: 174.0 ± 6.01 cm; body mass: 69.1 ± 6.4 kg; body mass index: 22.8 ± 1.6 kg/m^2^) were analyzed (Figure 2). The demographic information of participants can be found in Table 2. The eligibility criteria for the current study were: (i) soccer players with an experience of 5 years or above; (ii) have a history of at least two years of strength and conditioning training for soccer; and (iii) adherence rate of 85% of more during intervention; (iv) participated in the baseline and post-intervention assessments; (v) not being injured in the month before the start of the experimental period and not being injured during the experimental period; (vi) not taking anabolic steroids and hormonal precursors in the previous year and during the intervention; and (vii) the players did not take any drugs during the assessments and intervention period. The exclusion criteria were: (i) having illness or injury during the period of the study (ii) does not have clearance for performing resistance training; (iii) absence of more than 15% of the intervention sessions during the training period; (iv) participants should also not have used anabolic steroids or hormonal precursors in the previous year. The participants were informed about the study, its risks, and its benefits. After their verbal agreement, they signed a free consent form. The study followed the ethical standards for the study of humans as recommended by the Declaration of Helsinki.

### 2.4. Testing Procedures and Context

The evaluations of this study occurred before and after the intervention in the early season. In both moments, they had 24 h rest before assessments. For each of the week of assessments, the tests were organized by three days, interspaced by 24 h (Table 1). The mean air temperature was 24 °C all the assessments occurred between 3 and 5 p.m. Additionally, all the players were in the dormitory during this period and had the same sleep and nutrition and the training sessions lasted between 90 and 100 min. Players were introduced with test protocols and divided into two groups. Anthropometric measurements, blood sampling tests and the running-based anaerobic sprint test (RAST) were performed in the first test session. The linear print at 10, 20, 30 m and the change-of-direction (COD) tests were performed in the second day of assessments. The Yo-Yo Intermittent Recovery Test—level 1 (YYIRT1) was performed in the third day of assessments. The players’ warm-up protocol was performed on evaluation days including 5 min of jogging and 10 min including dynamic stretching exercises.

Both groups practiced for 5 sessions each week; the group performed HIRT training for 3 sessions and performed 2 sessions of technique and tactics exercises, but the control group performed techniques and tactics exercises every 5 sessions. The training sessions of each session consisted of 5 to 10 min of general warm-up and 20 min including specific warm-up, and immediately after the warm-up, the players performed the main exercises and in the third stage of training the players performed 10 to 15 min cooling, including soft runs and stretching movements. Players played 2 friendly matches during the study.

### 2.5. Anthropometry

The anthropometry assessment occurred about 3 h after the last meal, in a private room with an acclimatized temperature of 23 °C. The stature of the players was taken with them using sports shorts, t-shirts and without the use of shoes and socks. A stadiometer with an accuracy error of 0.1 cm was used to measure the stature (SECA 213, Hamburg, Germany). The body mass was tested using a digital flat scale (SECA 803, Hamburg, Germany) with an error of 0.1 kg. 

### 2.6. Blood Sampling

The blood sampling was performed in a laboratory during the morning period. The samples were taken in overnight fasting from 8 to 10 a.m. Players were positioned in a sitting position. Samples were collected from the antecubital vein into 15-mL vacutainer tuber. A 5 mL blood sample was centrifuged at 1500 rpm for 10 min at +4 °C. The process was performed independently by a certified laboratory. Malondialdehyde (MDA), Total superoxide dismutase (SOD), Glutathione assay (GSH), growth hormone (GH), cortisol and testosterone levels were determined in pre and post assessment periods. The MDA was assessed in a Libro S22 spectrophotometer (Biochrom, Cambridge UK). The GSH was analyzed in an ELISA plate reader (BIORAD). The cortisol and testosterone were analyzed using an Auto Chemistry Analyzer BM-100 (BioMaxima, S.A., Lublin, Poland). 

### 2.7. Running-Based Anaerobic Sprint Test (RAST)

The RAST test was implemented to test the ability to perform repeated sprints. The test consists of performing six consecutive line 35 m maximal runs, interspaced by 10 s of rest period [35]. The test was performed in a natural turf. The player started each run with the same preferred leg, using a split position. The sprint time was measured using two pairs of photoelectric cells (DSI, DANSH SALAR IRANIYAN, IRAN), positioned in 70 cm from the floor. The mean sprint time of the six runs were calculated as used as the main RAST outcome for further data treatment. 

### 2.8. Linear Sprint

For linear sprint tests 10, 20, 30 that all three were taken together, speed gate (Made in Iran) was used. The starting line was also marked 30 cm behind the gate, players were asked to use the preferable foot to start the Test at their own discretion. Each test was taken twice from the players and each time they had 2 min of inactive rest and the best record was recorded at the end.

### 2.9. Change-of-Direction Test 

The arrowhead test was employed accordingly to the protocol presented in a previous work [36]. Players with one foot on the start line and the other foot behind the starting line are on standby and with the command starting the speed ban towards the point and then with turns to marker B and then runs towards marker C and runs, the starting point once from the side Right and once from the left and for 5 min between each of them they performed passive rest and this test was taken twice and at the end the best record was recorded.

### 2.10. The Yo-Yo Intermittent Recovery Test—Level 1 (YYIRT)

In this test, players run between two cones with a distance of 20 m and then with each round of rounds, they perform an active rest between two cones with a distance of 5 m for 10 s, and then with each round of going back, the subject’s speed is added. The protocol was implemented based on the original one previously published [37]. The speed of running and rest time are controlled by the sound of the beep. If the tester does not reach the finish line before the beep is sounded, he will be warned and removed after two warnings. This test will be finished until the person can do so within a certain period of time, and the record of the person (distance covered in meters) is recorded and we calculate his aerobic power (VO_2_max) using previously published equation [38].

### 2.11. Training Intervention

These 8 weeks of the training program were performed in early season. Before the experimental study begin, all the players performed the same number and training sessions. The training process before this experimental approach was conducted by the coaches and consisted of field-based training sessions namely presenting periods of strength and conditioning emphasis, technical/tactical sessions, and formal matches. Usually, five training sessions per week, from those two sessions are dedicated to endurance training, one to speed and one to strength, and one to recovery (tapering). In this study, the HIRT group performed 3 HIRT sessions and 2 sessions of technique and tactics training, and the control group performed 5 sessions of technique and tactics training. The open field training sessions consisted of 10 min of general warm-up of jogging running and static stretching, 15 to 20 min of drill-based warm-up, then the main format exercises based on tactics and technical specificities of sport, and the last 10 to 15 min of training were cooling including soft running and stretching movements. The open field training was similar across the days regarding the structure and organization. The HIRT group performed 3 days a week (Table 3) and 2 with the control group performed techniques and tactics exercises. The HIRT group, while exercising, was asked to do all the exertion time at maximum intent aiming to stress as much as possible their participation ensuring maximal effort. Verbal encouragement was provided by the instructor as well as the sessions were supervised.

### 2.12. Statistical Procedures

Descriptive statistics is presented in mean and standard deviation. The normality and homogeneity of the sample was calculated using the Shapiro–Wilk and Levene’s tests. Considering the assumptions (*p* > 0.05), a mixed ANOVA was conducted to test the interaction between groups (HIRT vs. control) and time (baseline vs. post-intervention assessment). The partial eta squared ($\eta_{p}^{2}$) was executed to test the effect size of the mixed ANOVA. The independent t test was executed to test the between-groups analysis in both moments of assessment. The paired t-test was executed to test the within-group changes between assessments. The standardized effect size of Cohen (d) was calculated for t-tests. The magnitude of effect size (d) was considered based on the following thresholds [39]: 0.0–0.2, trivial; 0.2–0.5, small; 0.5–0.8, medium; >0.8, large. The statistical procedures were executed in the SPSS (version 28.0.0.0, IBM, Chicago, IL, USA) for a *p* < 0.05.

## 3. Results

Mixed ANOVA revealed significant interactions (group*time) on YYIRT1 (F = 13.402; *p* = 0.002; $\eta_{p}^{2\;}$= 0.456), sprint time at 10 m (F = 19.343; *p* < 0.001; $\eta_{p}^{2}\;$= 0.547), sprint time at 20 m (F = 7.133; *p* = 0.017; $\eta_{p}^{2}\;$= 0.308), sprint time at 30 m (F = 8.710; p = 0.009; $\eta_{p}^{2}\;$= 0.352), RAST (F = 35.427; *p* < 0.001; $\eta_{p}^{2}\;$= 0.689), VO2max (F = 13.104; *p* = 0.002; $\eta_{p}^{2}\;$= 0.450), change-of-direction (F = 38.170; *p* < 0.001; $\eta_{p}^{2}\;$= 0.705). Regarding hormonal, oxidative stress markers (MDA) and antioxidant markers in both groups mixed ANOVA revealed significant interactions (group*time) in cortisol (F = 12.566; *p* = 0.003; $\eta_{p}^{2}\;$= 0.440), GH (F = 8.184; *p* = 0.011; $\eta_{p}^{2}\;$= 0.338), testosterone (F = 9.780; *p* = 0.006; $\eta_{p}^{2}\;$= 0.379), SOD (F = 4.898; *p* = 0.042; $\eta_{p}^{2}$ = 0.234) and testosterone:cortisol ratio (F = 22.871; *p* < 0.001; $\eta_{p}^{2}\;$= 0.588). No significant interactions were found at MDA (F = 2.597; *p* = 0.127; $\eta_{p}^{2}\;$= 0.140), and GSH (F = 1.587; *p* = 0.226; $\eta_{p}^{2}\;$= 0.090).

Between-group variations (Table 4) executed by independent t test revealed no significant differences between HIRT and the ontrol group in the baseline assessments of physical fitness (*p* > 0.05). However, HIRT presented significant better results than control group on YYIRT (+142 m; *p* = 0.032; d = 1.108), VO2max (+1.20 mL/kg/min; *p* = 0.031; d = 1.114), 10 m sprint time (–0.06 s; *p* = 0.041; d = 1.049), 20 m sprint time (–0.10 s; *p* = 0.040; d = 1.053), 30 m sprint time (–0.24 s; *p* = 0.044; d = 1.028), RAST (–0.12 s; *p* = 0.013; d = 1.311), and COD (–0.37 s; *p* = 0.031; d = 1.175) after the intervention period. Regarding the biomarkers, between-group variations executed by independent t test revealed no significant differences between HIRT and control group in the baseline assessments of oxidative stress and antioxidant markers and hormonal levels (*p* > 0.05). However, HIRT presented significant smaller values of cortisol than control group (–62.11 mcg/dL; *p* = 0.031; d = 1.115), whereas it revealed significant greater testosterone (+1.23 ng/dL; *p* = 0.009; d = 1.395), testosterone:cortisol ratio (0.94 T:C ratio; *p* < 0.001; d = 1.693) and SOD (+2.91 ng/mg Hb; *p* = 0.035 d = 1.086) than control group.

**Figure 3:**
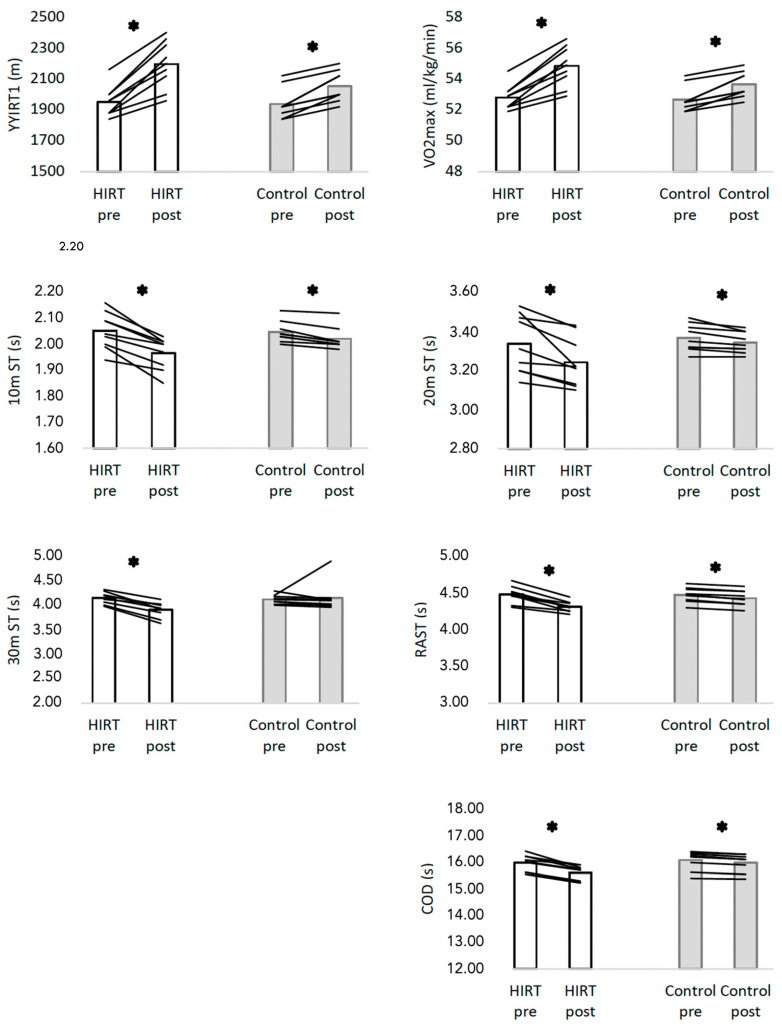
Descriptive statistics of physical fitness in both groups in pre- and post-intervention. HIRT: high-intensity resistance training; YYIRT1: The yo-yo intermittent recovery test level 1; ST: sprint time; RAST: running-based anaerobic sprint test; VO2max: maximal oxygen uptake; COD: change-of-direction time; * significant different at *p* < 0.05 (within-group).

The within group analysis revealed that the HIRT (Figure 3) group significantly improved the YYIRT1 (+12.5%; *p* < 0.001; d = 2.703), VO2max (+3.9%; *p* < 0.001; d = 2.684), 10 m sprint time (–3.9%; *p* < 0.001; d = 2.219), 20 m sprint time (–3.0%; *p* = 0.006; d = 1.239), 30 m sprint time (–5.8%; *p* < 0.001; d = 2.803), RAST (–3.8%; *p* < 0.001; d = 2.796) and COD (–2.3%; *p* < 0.001; d = 2.948).

The control group (Figure 3) also significantly changed YYIRT1 (*p* < 0.001; d = 2.118), VO2max (*p* < 0.001; d = 2.073), 10 m sprint time (*p* < 0.001; d = 2.177), 20 m sprint time (*p* = 0.006; d = 1.182), RAST (*p* < 0.001; d = 3.570) and COD (*p* < 0.001; d = 2.618. No significant changes were found at 30 m sprint time (*p* = 0.754; d = 0.108).

**Figure 4:**
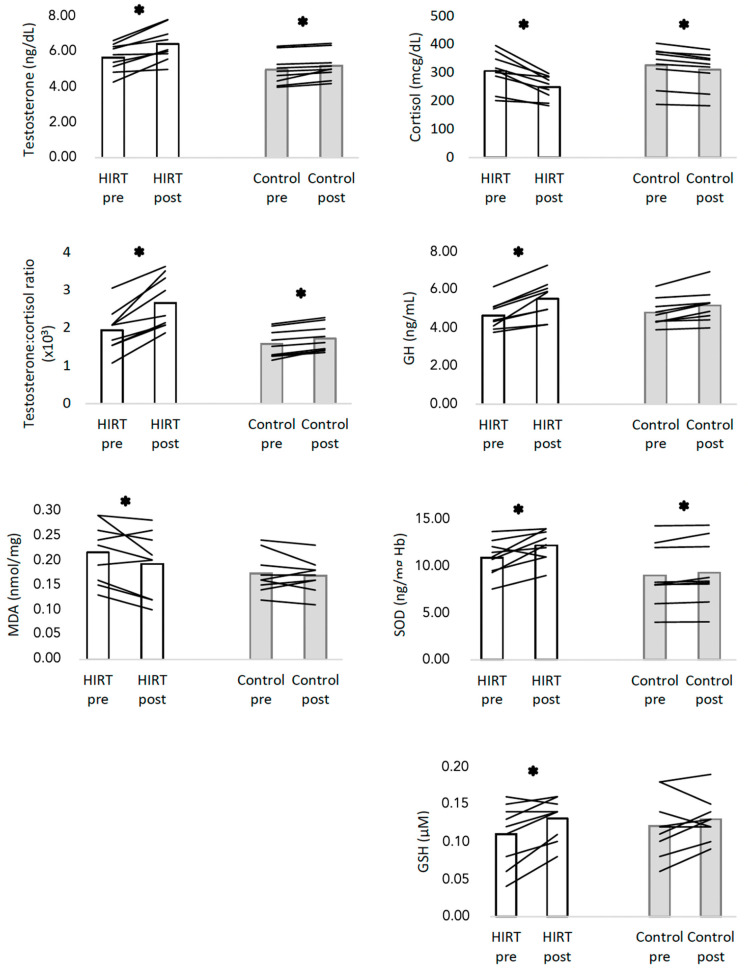
Descriptive statistics of hematological and biochemical parameters in both groups in pre- and post-intervention. HIRT: high-intensity resistance training; GH: growth hormone; SOD: Superoxide Dismutase; MDA: Malondialdehyde; GSH: Glutathione; * significant different at *p* < 0.05 (within-group).

The within group analysis (Figure 4) performed with paired t-test revealed that the HIRT group significantly reduced the levels of cortisol (–18.6%; *p* < 0.001; d = 1.653) and MDA (–9.5%; *p* = 0.021; d = 0.808), whereas it significantly increased the GH (+18.6%; *p* < 0.001; d = 1.882), testosterone (+13.6%; *p* < 0.001; d = 1.567), testosterone:cortisol ratio (0.72 T:C ratio; *p* < 0.001; d = 2.027), SOD (+12.0%; *p* = 0.009; d = 0.985) and GSH (+18.2%; *p* = 0.005; d = 1.111).

The control group (Figure 4) also significantly changed cortisol (*p* < 0.001; d = 2.316), GH (*p* = 0.002; d = 1.455), testosterone (*p* < 0.001; d = 1.567), testosterone:cortisol ratio (*p* < 0.001; d = 2.487), SOD (*p* = 0.040; d = 0.361). No significant changes were found in control ground considering the measure of MDA (*p* = 0.545; d = 0.020) and GSH (*p* = 0.261; d = 0.022).

## 4. Discussion

The purpose of this research was to analyze the effects of HIRT on the YYIRT, 10-, 20-, 30 m ST, RAST, COD, cortisol, testosterone, GH, SOD, MDA and GSH in adult male soccer players. The main findings showed that HIRT presented significant better results than control group in all physical fitness tests after the intervention period. In addition, the within group analysis revealed that both HIRT and control groups significantly improved all physical fitness tests with better results in the HIRT group. Furthermore, HIRT group significantly reduced the cortisol and MDA and increased GH, testosterone, SOD and GSH.

The HIRT had a significant beneficial effect on physical fitness. On one hand this information is extremely relevant because the HIRT protocol seems to be the first applied in adult male soccer players. On the other hand, it makes difficult to support it with the literature due to the scarce research in athletes as previously suggested by Hermassi et al. [25] because the majority of the studies with this type of intervention was applied in CrossFit training or amateur soccer [22,26,27,29,30,31,40]. In addition, the different physical fitness tests used in this study (YYIRT, 10-, 20-, 30 m ST, RAST, COD) were also different from others previously applied [22,25,26,27,29,30,31,40] and no other studies with soccer player and with the same type of protocol were found.

The YYIRT is a test with a higher aerobic component [38] whereas the remaining tests 10-, 20-, 30 m ST, RAST, COD) are related to anaerobic power and change of direction. Considering aerobic component, our findings seem to be in line with previous studies that showed improvements through HIRT [29,40,41,42]. Nonetheless, it is important to reinforce that different tests were used when compared to the present study, namely, graded exercise [29,40,41] or continuous tests [42]. We have implemented the YYIRT as the aerobic capacity test since is of intermittent nature following the specificity of the competitive soccer match [43]. The improvement caused by HIRT can be related to the oxidative stress promoted by the short periods of high-intensity effort performed repeatedly which will cause an adaptation in the cardiorespiratory system and the muscular structure (e.g., mitochondria) [44]. 

The anaerobic tests that analyzed sprint ability (10-, 20-, 30 m ST) also improved with the present training protocol which is corroborated by some studies [29,40], although once again different test such Wingate were used to access this component which is completely different from the tests used in the present study. The importance of sprinting in soccer is related to decisive moments during a match such escaping from an opponent player, reaching a free zone to shoot, or making a decisive pass [17]. In fact, sprints of 15 m Sprint [45] and 30 m Sprint [46] were previously reported as determinant to select under-17 soccer players. Since HIRT targets the muscle power, it is expected that improvements in sprint can be observed namely because is associated with muscle power and strength [47]. 

The repeated sprint ability measured by RAST also improved through HIRT. Previous research highlighted the importance of this ability in soccer players [48], since matches requires several acceleration, deceleration, and multi-directional changes [49] which reinforces the importance of such improvements. A study in handball players also revealed improvements in repeated sprint ability through HIRT intervention [25]. In the same line with previous results, COD ability also increased in this study. Such as repeated sprint, COD ability is also extremely important during matches because several sprint and stop, as well as repeated COD actions are constant abilities and reactions to other actions developed in the match [50,51]. The repeated sprint ability was also improved by the HIRT which can be caused by the improvement in aerobic capacity (which explain a reduction in the decrements of sprints) and also for the improvement in the sprint which positively affects the repeated sprint ability in early stages of the test [52].

Although the control group presented within-group improvements in physical fitness parameters, the post-assessment revealed that HIRT was significantly better than the control. Over the regular season, soccer players typically develop physical qualities such as aerobic fitness, anaerobic fitness, and muscular fitness possibly induced by field-based training. In fact, previous studies reveal improvements during the season in those parameters [53,54]. This is related to the physiological and locomotor stimulus associated with the specificity of the sport. It is expected that the regular training process may produce improvements in the main physical determinants of soccer players. However, the HIRT group was significantly better in improving these parameters suggesting that can be an interesting approach to complement the regular field-based training. In fact, some of the issues of the regular training are the heterogeneity of the stimulus, since some of the specific drill-based exercises are position-based which will conduct to natural variances between players. Using a standardized stimulus as HIRT, some players will possibly improve with a more stable stimulus being provided.

Physical effort and various forms of training, including resistance interval training, results in disbalance of metabolic processes, causing metabolic stress and strong autonomic response in the body. One of the elements of metabolic stress is the stimulation of the hormonal axes, including the hypothalamic–pituitary–adrenal axis (HPA axis), hypothalamic–pituitary–gonadal axis (HPG) [55] and others. The activation of HPA axis leads to the secretion of various anabolic and catabolic hormones and induces cellular oxidative stress [56,57]. The main stress and catabolic hormone is cortisol. It influences many body tissues involved in the adaptation processes during exercise. The tolerance of body adaptive mechanisms depends on the training period, the health body’s stage, preparation to withstand high training loads and its current emotional state [58]. 

One of the elements of metabolic stress is the load on the immune mechanisms and oxidative stress [59]. The negative effect of oxidative stress and the production of oxygen free radicals (ROS) is disruption of permeability and damage of the cell membrane structure of skeletal muscle fibers and cells of other tissues. It may results in oxidative modifications that disrupt the biological functions of organs [60]. ROS and oxidative stress underlie fatigue processes, especially overtraining the body, leading to an imbalance of anabolic and catabolic processes, disturbances in reaction speed or motor performance [59], which is reflected in a disturbed ratio of testosterone to cortisol (the T/C). On the other hand, skeletal muscle fibers damaged as a result of oxidative stress undergo regeneration and muscle hypertrophy. The different steps in the regeneration and hypertrophy process stay under testosterone and growth hormone control.

In our study, cortisol levels in the HIRT group were lower after the intervention and compared to the control group. It is in accordance with Rimmele et al. [61] results. Authors reported that elite athletes showed a lower reaction for cortisol, blood pressure, and heart rate compared to untrained men in response to acute psychosocial stress. In our study we also observed higher testosterone and GH levels after the training intervention and compared to the control group (GH, not significant). This means that training had a positive effect on synthesis processes and did not disturb the balance between anabolic-catabolic processes. According to Kraemer and Ratamess [62], anabolic hormones such as testosterone and or GH superfamily are elevated within 15–30 min after resistance exercise. High volume exercises as well as moderate and high in intensity, using short rest intervals and stressing a large muscle mass, tend to produce the greatest acute hormonal elevations than low-volume, high-intensity with long rest intervals. 

The balance of antioxidant/pro-oxidant processes in the body is related to intracellular defense mechanisms against ROS reactions. These mechanisms include reactions of antioxidant enzymes, i.e., catalase, glutathione peroxidase, superoxide dismutase or glutathione. The main role of cellular antioxidant defense is to scavenge the ROS. The activity of antioxidant enzymes in athletes blood depends on the degree of adaptation of the body to exercise/training.

In our study, we have reported lower levels of MDA in HIRT group after an innervation and higher levels of antioxidant markers—superoxide dismutase (SOD) and reduced glutathione (GSH). It means that the redox balance has been moved on antioxidant activity. This process can be induced by training stimulus [63]. That is characterized for adaptation to training load and of preseason and early season period. Taking into account of the hormones dynamic after the training intervention and prooxidant/antioxidant markers, we could say that changes reflect the positive direction characterized the proper training load, which stimulates anabolic processes [64]. This is essential at the preparation stage of the preseason period and the early season [65].

Despite the positive results described before, this study presents some limitations. The small sample of professional soccer players does not allow generalization of results for other populations or sports. Additionally, hemodynamic parameters lacked in the current research. They should be considered in future research. Additionally, other markers should be considered in future research such as 8-isoprostane to understand oxidative stress better. The period of analysis should also be considered in future interventions, namely analyzing the antioxidative factors more regularly. Another limitation is related with physical tests. some studies suggested that strength of the lower limbs should be accessed through separate tests (i.e., linear and non-linear sprints, COD, jump ability) [66,67,68]. While we applied different linear and non-linear sprints as well as COD, we failed in using jump or strength tests which should be included in future research to amplify results. Furthermore, we could not find other similar training protocols in the literature which requires mores studies to confirm our results. The training intensity was only controlled by asking athletes to perform all exercises as fast as possible with no measure of intensity applied which should be included in future studies. Moreover, the training load was not monitored in open field sessions which should be implemented in future research.

Even though this study presents several strengths and practical applications, several improvements in different physical fitness, hormonal and antioxidant factors were found when performing three regular soccer training sessions, plus three HIRT sessions compared with only three regular soccer training sessions. Such findings are more relevant because no research was found in soccer young adult athletes and reinforces the importance of additional resistance training to develop physical and physiological capacities of soccer players. Moreover, this study provides a propose of HIRT protocol that can be replicated by future studies during the pre-season period. In fact, future research can test the application of HIRT in some periods of the in-season with low training intensity (i.e., where a low number of matches may occur). As observed in previous studies, HIRT can develop several capacities such aerobic power, anaerobic capacity, strength/resistance in non-athletes [23,29]. Similar results were found in handball athletes, but more studies are needed [25].

## 5. Conclusions

This study suggests that HIRT can be an effective approach for improving physical fitness and modulating endocrine and antioxidant while used as a complementary approach for the in-field training session of soccer players. Although there were limitations to the study, it was possible to observe that, from a physical and physiological point of view, the introduction to HIRT positively affected the aerobic capacity, sprint, and the ability to repeatedly perform sprints. These improvements were significantly greater than those obtained in the control group after the intervention. Additionally, those experiencing HIRT were significantly beneficiated on greater levels of testosterone and SOD, while decreasing cortisol.

## Figures and Tables

**Table 1:** Timeline of the study.

Days	1st Week	2nd Week	3rd Week	4th Week	5th Week	6th Week	7th Week	8th Week	9th Week	10th Week
Sunday	Resting day	Resting day	Resting day	Resting day	Resting day	Resting day	Resting day	Resting day	Resting day	Resting day
Monday	Test	HIRT Training	HIRT Training	HIRT Training	HIRT Training	HIRT Training	HIRT Training	HIRT Training	HIRT Training	Test
Tuesday	Resting day	Tac/Tec Training	Tac/Tec Training	Tac/Tec Training	Tac/Tec Training	Tac/Tec Training	Tac/Tec Training	Tac/Tec Training	Tac/Tec Training	Resting day
Wednesday	Test	HIRT Training	HIRT Training	HIRT Training	HIRT Training	HIRT Training	HIRT Training	HIRT Training	HIRT Training	Test
Thursday	Resting day	Resting day	Resting day	Resting day	Resting day	Resting day	Resting day	Resting day	Resting day	Resting day
Friday	Test	HIRT Training	HIRT Training	HIRT Training	HIRT Training	HIRT Training	HIRT Training	HIRT Training	HIRT Training	Test
Saturday	Resting day	Tac/Tec Training	Tac/Tec Training	Match day	Tac/Tec Training	Tac/Tec Training	Match day	Tac/Tec Training	Tac/Tec Training	Resting day

HIRT: high-intensity resistance training; Tac/tec: tactical/technical training session.

**Table 2:** Demographic information of the participants.

Outcomes	HIRT Group	Control Group	Total
Participants (*n*)	9	9	18
Age (years)	20.3 ± 0.6	20.4 ± 0.7	20.4 ± 0.7
Experience (years)	6.0 ± 2.5	5.3 ± 2.1	5.6 ± 2.3
Stature (cm)	173.0 ± 6.1	175.0 ± 6.1	174.0 ± 6.0
Body mass (kg)	69.7 ± 6.5	68.6 ± 6.5	69.1 ± 6.4
Body mass index (kg/m^2^)	23.2 ± 1.4	22.4 ± 1.7	22.8 ± 1.6
Defenders (*n*)	4	3	8
Midfielders (*n*)	3	4	8
Attackers (*n*)	2	2	4
Adherence (%)	100	100	100

HIRT: high-intensity resistance training.

**Table 3:** Training plan.

*n*	Name	Set		Rep	Rest	Rest between Move
1	Bodyweight double-leg squat	2	3	12	1 min	1 min
2	Bodyweight alternating lunge	2nd to 5th week	2nd to 5th week	12	1 min	1 min
3	Alternating split jump			12	1 min	1 min
4	Squat jump			12	1 min	1 min
5	Agility ladder split step			10s	1 min	1 min
6	agility ladder lateral rotational jump			10	1 min	1 min
7	Bodyweight push-up			12	1 min	1 min
8	MB single-arm push-off			12	1 min	1 min
9	MB crossover push-up			12	1 min	1 min
10	MB overhead slam			12	1 min	1 min

**Table 4:** Descriptive statistics (mean and standard deviation) of physical fitness outcomes in the baseline and post-intervention and comparisons between-groups in both moments.

Outcome	HIRT Baseline (Mean ± SD)	Control Baseline (Mean ± SD)	HIRT vs. Control (Baseline) | *p*-Value and d (Effect Size)	HIRT Post-Intervention (Mean ± SD)	Control Post-Intervention (Mean ± SD)	HIRT vs. Control (Post-Intervention) | *p*-Value and d (Effect Size)
YYIRT1 (m)	1951.1 ± 97.5	1937.8 ± 98.2	*p* = 0.776; d = 0.136	2195.6 ± 152.9	2053.3 ± 98.0	*p* = 0.032 *; d = 1.108
VO2max (mL/kg/min)	52.80 ± 0.80	52.68 ± 0.82	*p* = 0.753; d = 0.151	54.84 ± 1.28	53.64 ± 0.82	*p* = 0.031 *; d = 1.114
10 m ST (s)	2.05 ± 0.07	2.05 ± 0.04	*p* = 0.873; d = 0.077	1.97 ± 0.06	2.02 ± 0.04	*p* = 0.041 *; d = 1.049
20 m ST (s)	3.34 ± 0.15	3.37 ± 0.07	*p* = 0.594; d = 0.256	3.24 ± 0.12	3.34 ± 0.05	*p* = 0.040 *; d = 1.053
30 m ST (s)	4.15 ± 0.12	4.12 ± 0.09	*p* = 0.605; d = 0.249	3.91 ± 0.16	4.15 ± 0.29	*p* = 0.044 *; d = 1.028
RAST (s)	4.48 ± 0.11	4.48 ± 0.10	*p* = 0.920; d = 0.048	4.31 ± 0.07	4.43 ± 0.10	*p* = 0.013 *; d = 1.311
COD (s)	15.98 ± 0.31	16.09 ± 0.35	*p* = 0.753; d = 0.335	15.62 ± 0.28	15.99 ± 0.33	*p* = 0.031 *; d = 1.175
Cortisol (mcg/dL)	307.3 ± 65.8	327.7 ± 71.1	*p* = 0.536; d = 0.298	250.0 ± 42.4	312.1 ± 66.4	*p* = 0.031 *; d = 1.115
GH (ng/mL)	4.68 ± 0.76	4.83 ± 0.72	*p* = 0.673; d = 0.202	5.55 ± 1.03	5.20 ± 0.86	*p* = 0.439; d = 0.374
Testosterone (ng/dL)	5.68 ± 0.80	5.00 ± 0.85	*p* = 0.099; d = 0.826	6.45 ± 0.97	5.22 ± 0.79	*p* = 0.009 *; d = 1.395
T:C ratio (x10^3^)	1.95 ± 0.58	1.58 ± 0.37	*p* = 0.126; d = 0.760	2.67 ± 0.70	1.73 ± 0.36	*p* = 0.004; d = 1.693
MDA (nmol/mL)	0.21 ± 0.06	0.17 ± 0.04	*p* = 0.106; d = 0.807	0.19 ± 0.07	0.17 ± 0.03	*p* = 0.353; d = 0.451
SOD (ng/mg Hb)	10.91 ± 1.89	9.02 ± 3.29	*p* = 0.155; d = 0.704	12.22 ± 1.68	9.31 ± 3.39	*p* = 0.035 *; d = 1.086
GSH (μM)	0.11 ± 0.04	0.12 ± 0.04	*p* = 0.575; d = 0.270	0.13 ± 0.03	0.13 ± 0.03	*p* = 0.935; d = 0.039

SD: standard-deviation; HIRT: high-intensity resistance training; YYIRT1: The yo-yo intermittent recovery test level 1; ST: sprint time; RAST: running-based anaerobic sprint test; VO_2_max: maximal oxygen uptake; COD: change-of-direction time; HIRT: high-intensity resistance training; GH: growth hormone; SOD: Superoxide Dismutase; MDA: Malondialdehyde; GSH: Glutathione; T:C ratio: testosterone:cortisol ratio; *: significant different at *p* < 0.05.

## Data Availability

Not applicable.
